# Rapid expansion of lymphogranuloma venereum infections with fast diversification and spread of *Chlamydia trachomatis* L genovariants

**DOI:** 10.1128/spectrum.02855-23

**Published:** 2023-12-14

**Authors:** Laura Martínez-García, Eva Orviz, José María González-Alba, Alicia Comunión, Teresa Puerta, María Mateo, Matilde Sánchez-Conde, María Concepción Rodríguez-Jiménez, Mario Rodríguez-Domínguez, Francisco Javier Bru-Gorraiz, Jorge del Romero, Rafael Cantón, Juan Carlos Galán

**Affiliations:** 1 Servicio de Microbiología, Hospital Universitario Ramón y Cajal and Instituto Ramón y Cajal de Investigación Sanitaria (IRYCIS), Madrid, Spain; 2 Centro de Investigación Biomédica en Red en Epidemiología y Salud Pública (CIBERESP), Madrid, Spain; 3 Centro Sanitario Sandoval, Hospital Clínico San Carlos, Instituto de Investigación Sanitaria San Carlos (IdISSC), Madrid, Spain; 4 Servicio de Microbiología, Hospital Universitario Central de Asturias, Instituto de Investigación Sanitaria del Principado de Asturias (ISPA), Oviedo, Spain; 5 Servicio de ITS-Dermatología, Centro Montesa, Madrid, Spain; 6 Servicio de Microbiología, Hospital Central de la Defensa Gómez-Ulla, Madrid, Spain; 7 Servicio de Enfermedades Infecciosas, Hospital Universitario Ramón y Cajal and Instituto Ramón y Cajal de Investigación Sanitaria (IRYCIS), Madrid, Spain; 8 Centro de Investigación Biomédica en Red en Enfermedades Infecciosas (CIBERINFEC), Madrid, Spain; Michigan State University, East Lansing, Michigan, USA

**Keywords:** sexually transmitted infections, lymphogranuloma venereum, molecular epidemiology, genovariants

## Abstract

Invasive genotypes of *Chlamydia trachomatis* (L1–L3) are responsible for lymphogranuloma venereum (LGV), which, in industrialized countries, is prevalent among men who have sex with men. Our aim was to analyze the changing trends and diversification of LGV genovariants detected in Madrid, Spain. The study was launched at a public community healthcare center that has specialized in sexually transmitted infections (STIs) for 10 years (STI1). Screening rates have improved at this center, but LGV diagnoses have drastically increased, especially since 2016. We therefore extended the LGV diagnosis to another public community healthcare center that specializes in STIs (STI2), as well as to two general tertiary hospitals in Madrid from 2017 to 2019. Overall, between 2010 and 2019, 10,833 cases of *C. trachomatis* infection were detected among 121,955 screened samples with 1,253 LGV cases. The molecular characterization of L genotypes identified two main genovariants (ompA-L2 and ompA-L2b) in 2010–2014. However, three new successful genovariants (ompA-L2bV1, ompA-L2bV4, and ompA-L2bV7) emerged in 2016–2019. In recent years, 3.9% (31/803) of recombinant forms of ompA were detected between ompA-L2 and ompA-D. From the clinical and epidemiological standpoints, the patients infected by the ompA-L2 and ompA-L2bV1 genovariants were more frequently associated with asymptomatic infections and HIV (*P* = 0.001), while ompA-L2b and ompA-L2bV4 were more frequently associated with concomitant (*P* < 0.02) and previous (*P* = 0.003) STI infections, suggesting distinct sexual networks. These findings suggest a growing trend toward higher transmission rates in the population along with an increasingly complex epidemiological scenario due to the selection of LGV genovariants through mutation and recombination.

**IMPORTANCE:**

Numerous international organizations, including the World Health Organization, have been drawing attention to the global increase in sexually transmitted infections. Twenty years ago, lymphogranuloma venereum (LGV) was mainly considered a tropical disease; in recent decades, however, LGV has been increasingly present in high-income countries. This increase has been linked to men who have sex with men who participate in highly interconnected sexual networks, leading to a rapid spread of LGV. This study focuses on the spread of LGV, presenting the largest time series of LGV prevalence in Spain, which includes more than a thousand diagnosed cases in one large city. The number of LGV cases diagnosed was analyzed over time, and a selection of strains was subjected to molecular genotyping. The results indicate that the LGV epidemic is gradually evolving toward an increasingly complex diversification due to the selection of successful genovariants that have emerged by mutation and recombination events, suggesting that we are moving toward an unpredictable scenario.

## INTRODUCTION

In 2019, the World Health Organization (WHO) reported that there were more than one million new cases of curable sexually transmitted infections (STIs) every day (1) and that this number was increasing. In 2017–2019, the American and European Annual Epidemiological Reports showed a significant increase in the overall notification rates for infections caused by *Neisseria gonorrhoeae* (11% and 31% in the US and Europe, respectively), *Chlamydia trachomatis* (5.5% and 6.7%), and *Treponema pallidum* (27% and 3.5%) (2, 3), with the latest US data showing the trend continuing upward (2). Due to the concern regarding the worldwide increase in STIs, the 75th World Health Assembly redesigned a global health sector strategy by including HIV, hepatitis, and STIs in the same agenda for 2022–2030 (4). The latest 2019 European Annual Epidemiological Report for lymphogranuloma venereum (LGV) (infections caused by the L1–L3 genotypes of *C. trachomatis*) revealed the largest number of reported cases, confirming a 30% increase in the number of diagnoses in 2019 over the previous year (2,389 vs 1,989) (5). However, 86% of all European LGV diagnoses were reported by four countries with a well-implemented reporting system: the United Kingdom, France, the Netherlands, and Spain, implying uneven implementation of LGV testing and reporting (5); results suggest that the number of LGV infections in Europe might be underestimated. An integrated WHO surveillance strategy could, therefore, provide an opportunity to visualize STIs in all national surveillance programs.

The current LGV epidemic, which is closely associated with men who have sex with men (MSM), started in the Netherlands in 2003 (6); however, cases soon began to appear in other European countries, North America, and Australia (7). This rapid spread of LGV was attributed to the emergence of a new L genotype known as the L2b variant (8), which was responsible for most (if not all) cases during the early years from 2003 (9). A number of European countries reported the circulation of non-*ompA*-L2b genovariants (10, 11) in the initial years; however, full genomic sequencing studies revealed that all these genovariants were derived from L2b (12). More recently, several studies have reported an increase in LGV diagnoses in asymptomatic patients (13), revealing that the spread of undiagnosed LGV was favoring the selection and dispersal of less virulent genovariants, which could contribute to a more complex epidemiological scenario characterized by the accumulation of genovariants that are difficult to detect without proactive diagnostic strategies. This conclusion was reinforced by the identification of recombinant variants between L genotypes (invasive genotypes) and non-L genotypes (non-invasive genotypes such as A-K), such as the highly virulent L2-D recombinant variant (14), a finding that has complicated the description of the epidemiological scenario and results from the risks associated with the selection of new variants with unforeseen effects on local epidemiology.

Few studies have previously analyzed the spread and diversification of the L2b genotype and genovariants over a long period (15, 16). In a previous LGV European collaborative study, Spain showed the highest diversity rate (12). With our study, we aimed to analyze the temporal evolution of LGV infections at four centers in Madrid representing distinct clinical settings and target populations and aimed to characterize the emergence, diversification, and spread of L2b genovariants.

## MATERIALS AND METHODS

### Collection of clinical samples

The samples were collected from patients who voluntarily attended one of the four participating health centers in the context of routine clinical care for a suspected STI as a result of high-risk sexual behavior (e.g., unprotected sex, a large number of sexual partners, and sexual encounters with unknown individuals). STI testing was provided to all individuals at risk of exposure, regardless of their symptoms. Urethral/cervical, rectal, and pharyngeal samples were taken depending on their sexual behaviors. All samples testing positive for *C. trachomatis* amplification (mostly rectal samples) were sent for genotyping to the coordinating laboratory in the Microbiology Department of Ramon y Cajal Hospital. The participating centers included two community healthcare centers that specialize in STIs [CS Sandoval (STI1) and CS Montesa (STI2)] and two tertiary general hospitals (Hospital Universitario Ramón y Cajal and Hospital Universitario Central de la Defensa Gómez Ulla). The patients attending STI1 are mainly MSM with a high number of sexual partners and/or take psychotropic drugs to enhance sexual activity, while STI2 serves an almost equal number of mostly heterosexual men and women (including female sex workers) and MSM. The hospitals serve the general population with a similar proportion of men and women and with various probabilities of high-risk sexual exposure. In the hospital setting, the samples were collected by the infectious disease hospital departments and primary care centers located in the urban health area covered by these hospitals. Samples were collected at STI1 between 2010 and 2019, whereas the sampling period for the other three centers was 2017–2019.

### Detection of the *C. trachomatis* strain in clinical samples

**Fig 1:**
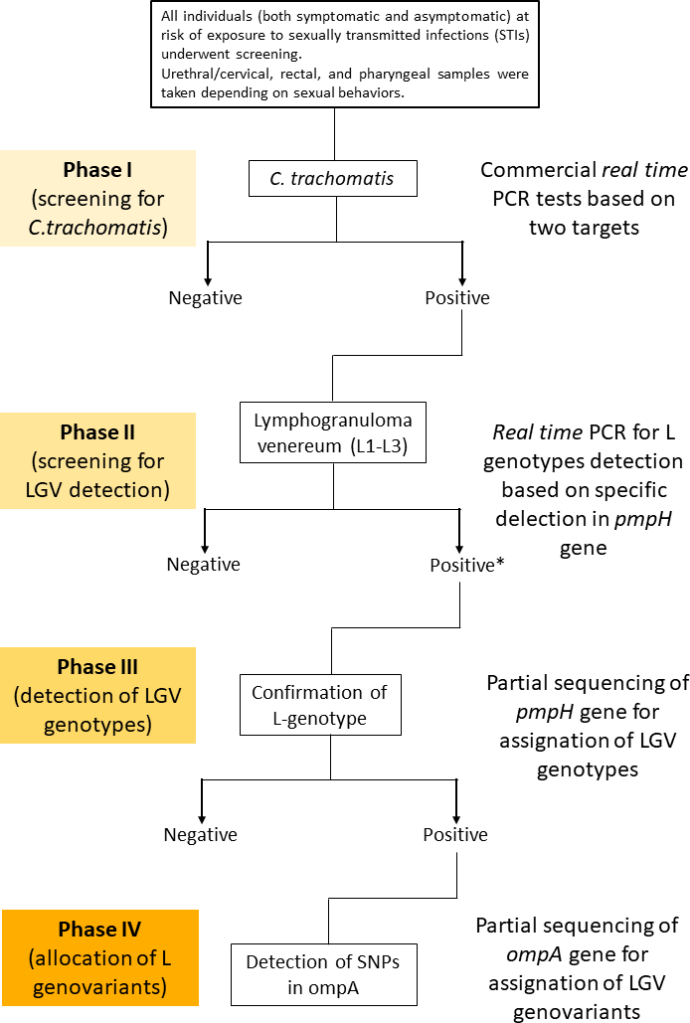
Flowchart of the experimental design. Phase I involves the screening for *C. trachomatis* using various commercial tests. Phase II consists of screening for LGV, detecting the 36-bp deletion in the *pmpH* gene, which is specific to L genotypes. Phase III involves confirmation of suspected L genotypes through the partial sequencing of the *pmpH* gene. This provides an accurate classification of L genotypes. Phase IV entails allocating genovariants based on partial sequencing of the *ompA* gene. Abbreviations for technical terms will be explained when introduced. *Not all *pmpH* genes were sequenced for the confirmation of suspected L genotypes.

*C. trachomatis* infections were detected at each participating center using commercially available tests as part of the daily routine in the context of STI screening and using various molecular platforms, including the Allplex STI Essential Assay (Seegene, Seoul, South Korea) in the Microbiology Department of University Hospital Ramón y Cajal, Cobas 4800 CT/NG (Roche Molecular Systems, Inc., Pleasanton, CA, USA) in the University Hospital Central de la Defensa Gómez Ulla, and Abbott m2000 RealTime PCR (Abbott Molecular Inc., Des Plaines, IL, USA) in STI1 and STI2 (phase I in Fig. 1). The detection of *C. trachomatis* using these commercial platforms was based on a cryptic plasmid (± ompA gene). The cycle threshold (Ct) for considering a sample positive or negative for *C. trachomatis* infection was defined by the manufacturer’s recommendations.

### Detection of LGV strains, genotyping, and characterization of genovariants

All samples that yielded a positive amplification for *C. trachomatis* were sent to the laboratory of the Microbiology Department of University Hospital Ramón y Cajal for genotyping, which then extracted the DNA from the samples using NucliSENS easyMAG (bioMerieux Inc., Durham, NC, USA) according to the manufacturer’s instructions. Detection of invasive L genotypes (those related to LGV disease) was performed using real-time PCR based on the 36-bp deletion on the *pmpH* gene (17), which is specific for invasive genotypes (L genotypes). A Ct value ≤35 was considered as positive amplification. This diagnosis strategy detects all L genotypes (L1–L3) (phase II in Fig. 1). A 404-bp *pmpH* fragment was sequenced by Sanger technique, and phylogenetic reconstructions based on *pmpH* gene provided confirmation of the L genotype, given that this gene differentiates genotypes according to disease type (trachoma, lymphogranuloma, and genital tract infections) (18) (phase III in Fig. 1). Once the L genotype was established, an 858-bp fragment of the *ompA* gene (encoding the major outer membrane protein) was sequenced by Sanger in all samples in which L genotypes were detected by sequencing or by real-time PCR. The genetic information obtained made it possible to identify rapid diversification because this gene is probably the most mutagenic (19) (phase IV in Fig. 1). The sequencing of *pmpH* and *ompA* has become a popular technique in LGV epidemiology. PmpH ensures accurate genotype assignment according to the known pathotypes, while *ompA* is subject to the most selective pressure (positive selection), with an overrepresentation of single-nucleotide polymorphisms, which offers a strategy for detecting diversification (see the Fig. 1 legend for more details).

The primers, probes, and conditions for all assays are shown in Table S1.

### Statistical analysis

The data were analyzed with the statistical program STATA 13.0 (StataCorp LP, TX, USA). To determine the association between genovariants with clinical and epidemiological variables, Pearson’s chi-squared test was performed. When the expected frequency was <5, Fisher’s exact test was employed.

### Phylogenetic analysis

The ClustalW program implemented in MEGA was employed to align the *ompA* sequences that were obtained along with all *C. trachomatis* sequences available in the GenBank database (www.ncbi.nlm.nih.gov). A nucleotide substitution model was selected for each gene using jModeltest 1.0 software (20), and maximum likelihood phylogenetic trees were constructed with PhyML 3.0 (21). To determine potential recombination events, the sequences were analyzed using the recombination detection program (RDP3v4.13) (22). Novel genovariants among L2b genotypes were defined using the “mean junction” method to build networks from recombination-free population tion (www.fluxus-technology.com).

### Clinical and epidemiological data

We retrieved clinical and epidemiological data from the participants’ medical records. The participating physicians reviewed, collected, and anonymized the relevant information such as age, sex, home country, signs and symptoms, HIV and hepatitis serological status, previous and concomitant STIs, and treatment. The patients were asked about their sexual history and drug use according to clinical practice. Only medical records from patients infected by the main genovariants (L2, L2b, L2bV1, and L2bV4) were reviewed. The study was approved by the Ethics Committee of University Hospital Ramón y Cajal (reference 012/17).

## RESULTS

### Changing trends of LGV diagnosis at STI1, the STI-specialized healthcare center mainly attended by MSM, in 2010–2019

During the study period, 8,325/85,268 (9.7%) clinical specimens tested positive for *C. trachomatis*. Of these, 1,171 (14.1%) were identified as belonging to the L genotypes (causing LGV) based on the 36-bp deletion in the *pmpH* gene (Fig. S1).

**Fig 2:**
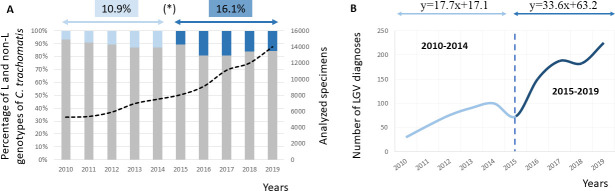
(**A**) Percentage of L (light and dark blue columns) and non-L (gray columns, which include A-K) genotypes detected with respect to all analyzed specimens in the STI1. The dashed black line represents the number of screened samples in the studied period. The dashed gray line represents the percentage of positive *C. trachomatis* samples with respect to the total number of screened samples. * Mean values (from 2010 to 2014 and 2016 to 2019) of L genotypes detected with respect to all identified cases of *C. trachomatis* infection. (**B**) Temporal evolution of lymphogranuloma venereum diagnoses detected in the specialized STI1 attended mainly by MSM (2010–2019). Two periods could be distinguished based on the annual detected L-genotype infections The slope of the curve was 1.9-fold higher in the second period (2016–2019) than in the first period (2010–2014), suggesting an acceleration in LGV diagnoses. In fact, although the number of LGV infections increased year after year, the magnitude of the increase progressively decreased with each passing year in the period 2010–2014: 80% (2010–2011), 40.7% (2011–2012), 19.7% (2012–2013), and 9.9% (2013–2014). In 2015, there was a notable decrease in the number of LGV diagnoses compared with the previous year (more samples screened but fewer positive results). Since 2015, the number of diagnoses has increased to 50% (2016–2014), 24% (2017–2016), and 23% (2019–2018) during the second period.

The number of samples screened for *C. trachomatis* increased from 5,276 in 2010 to 14,011 in 2019, representing a 2.7-fold increase over that 10-year period (Table 1). The improvement in screening rates resulted in an increase in the diagnosis of *C. trachomatis* infections from 437 in 2010 to 1,434 infections in 2019, representing a 3.3-fold increase over that period. L-genotype infections were also detected more frequently, from 30 cases in 2010 to 225 in 2019, representing a more than sevenfold increase (Table 1). In 2010–2014, the proportion of L-genotype infections among all positive *C. trachomatis* samples was 10.9% (351/3,232), whereas in 2016–2019, this proportion reached 16.1% (820/5,093), a statistically significant difference (*P* < 0.00001) (Fig. 2A). Two periods could be distinguished based on the annual detected L-genotype infections suggesting an acceleration in LGV diagnoses in the 2016–2019 period (a more detailed information is available in Fig. 2B).

**TABLE 1:** Changes in the screening and detection trends for sexually transmitted infections in the clinical settings analyzed

	Samples for CT screening			CT-positive samples			LGV-positive samples			LGV/*C. trachomatis*		
	2010/2017^*a*^	2019	Increase	2010/2017^*a*^	2019	Increase	2010/2017^*a*^	2019	Increase	2010/2017^*a*^	2019	Increase
STI1	5,276	14,011	2.7-fold	437	1,434	3.3-fold	30	225	7.5-fold	6.8%	15.7%	2.3
STI2	4,901	10,449	2.1-fold	301	662	2.2-fold	6	24	4.0-fold	2.0%	3.6%	1.8
Hospitals	2,980	4,477	1.5-fold	301	245	−1.2-fold	9	10	−1.1-fold	3%	3.1%	1.03

^*a*^ The period lasted 10 years, whereas in the other centers, the period lasted only 3 years (2017–2019).

### Expanded screening for L-genotype detection to the additional centers between 2017 and 2019, following an observed increase at STI1

**Fig 3:**
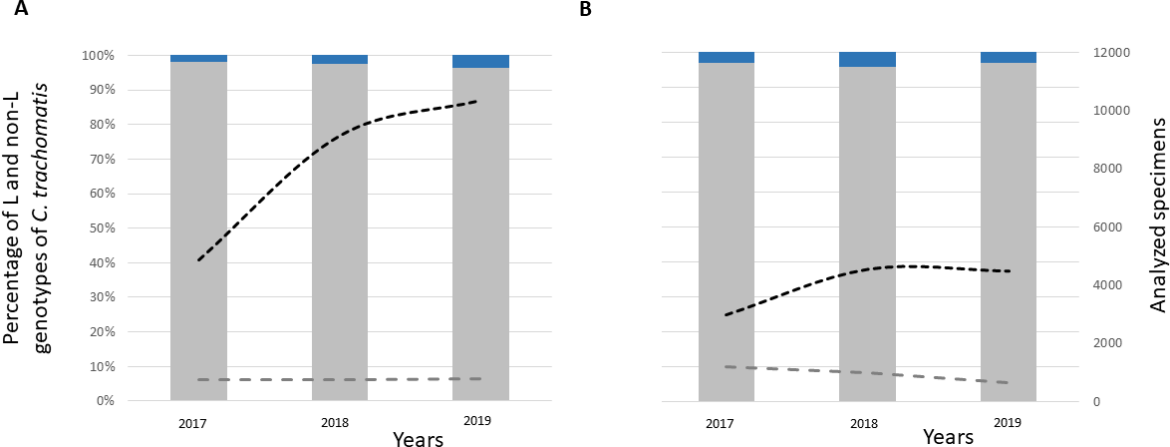
Percentage of L (dark blue columns) and non-L (gray columns) genotypes detected with respect to all analyzed specimens in the 2017–2019 period. (**A**) In the specialized STI2. (**B**) In the two hospitals. Detailed information is shown in Fig. 2.

Due to a shift in the trends observed at the STI1 center, which predominantly serves MSM, the search for L genotypes was extended in 2017 to three additional centers attending individuals with a wide range of exposure opportunities. In STI2, the number of samples tested over the 3-year period increased from 4,901 in 2017 to 10,449 in 2019, representing a 2.1-fold increase (Fig. 3A). The improvement in screening rates enabled the detection of 301 cases of *C. trachomatis* infection in 2017 and 662 infections in 2019, which represents a 2.2-fold increase and is identical to the increase in screening. As expected, L genotype detection also increased (from 6 cases in 2017 to 24 in 2019), but the increase in LGV infections (4.0-fold) outpaced the increase in screening rates (2.1-fold) (Table 1).

In the hospital setting, the number of samples analyzed increased from 2,980 to 4,477 in the final 3 years, which represents a modest 1.5-fold increase (Fig. 3B). Surprisingly, the number of *C. trachomatis* infections decreased from 301 to 245 (1.2-fold decrease), but the number of LGV diagnoses remained stable (9 and 10 in 2017 and 2019, respectively) (Table 1).

### Genotyping of L genovariants based on *pmpH* and *ompA* detected in all centers

Based on the real-time PCR results employed to infer the presence of L genotypes, the *pmpH* gene was also sequenced in 599 samples to confirm the correct assignation to the L genotype, with all sequenced strains identified as belonging to the L genotype. Once the L genotype was correctly identified, the *ompA* gene was amplified and sequenced in 803/1,244 samples (64.5%) (Table S2 shows the non-synonymous mutations). Sequencing of *ompA* provided insight into the *ompA* genovariants circulating in the population. Overall, *ompA*-L2 (accession number AM884176) and its evolved genovariants were found in 424/803 of the strains (52.8%), while *ompA*-L2b (accession number AM884177, characterized by a single mutation, N162S, with respect to *ompA*-L2) and its evolved genovariants were found in 348/803 (43.3%). Among the evolved genovariants, *ompA*-L2bV1 (accession number JX971936), which carries a non-synonymous mutation (L173I) with respect to *ompA*-L2b, and *ompA*-L2bV4 (accession number KU518892) were characterized by two non-synonymous mutations (A91T and H165N) in *ompA* relative to *ompA*-L2b. *ompA*-L2bV7 (accession number LR882815) was characterized by a non-synonymous mutation (S333N) in *ompA* relative to *ompA*-L2b. A genovariant characterized by the mutation Q75R was associated with both *ompA*-L2 and *ompA*-L2b (without specific assigned name).

Most mutations observed in *ompA* were detected in variable domains I–IV, which are surface-exposed domains involved in adhesion and immune response (21). One of the interesting findings was that the mutations identified during the initial period were different from those found in the subsequent period, except for the mutations linked to *ompA*-L2bV1, which were observed in both (Table S2).

### Dynamic temporal of genovariants and difference between periods

**Fig 4:**
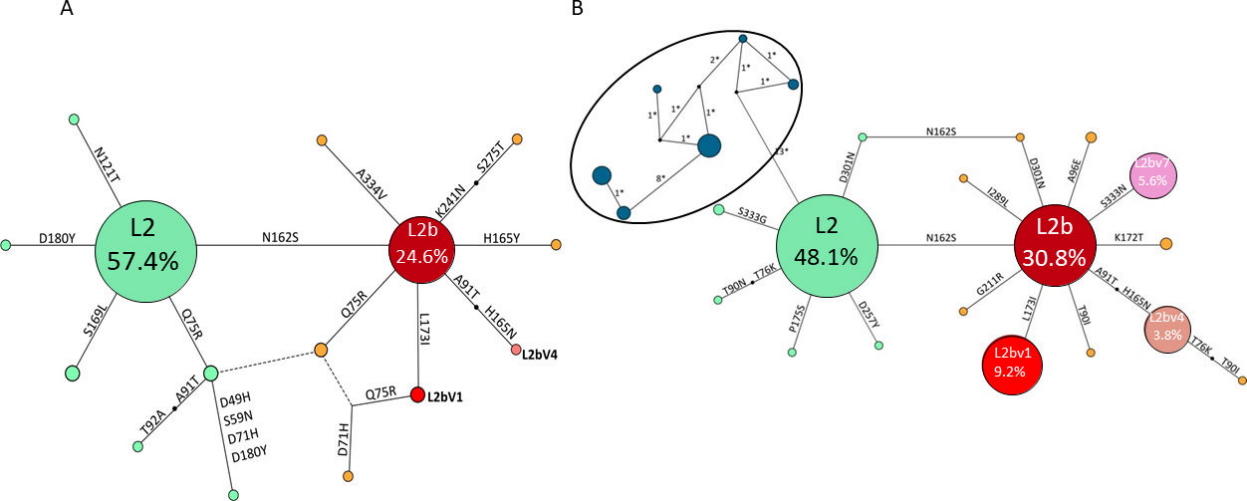
Phylogenetic network constructed using *ompA* sequences of lymphogranuloma venereum diagnoses. (**A**) The 2010–2014 period (*n* = 120). (**B**) The 2016–2019 period (*n* = 679). The numbers inside the circles correspond to percentages with respect to the sequenced samples. The numbers above the lines correspond to the number of non-synonymous changes. Blue circles correspond to *ompA*-recombinant variants between L2 and D genotypes. Moreover, 3/73 variants derived from L1 and 4/73 were *ompA*-variants non-related to L genotypes (2 D, 1 J, and 1 K, although *pmpH* sequencing allowed them to be classified as such). * Numerous samples from the 2010–2014 period were previously published by our group (10) or in an international collaborative study (12).

**Fig 5:**
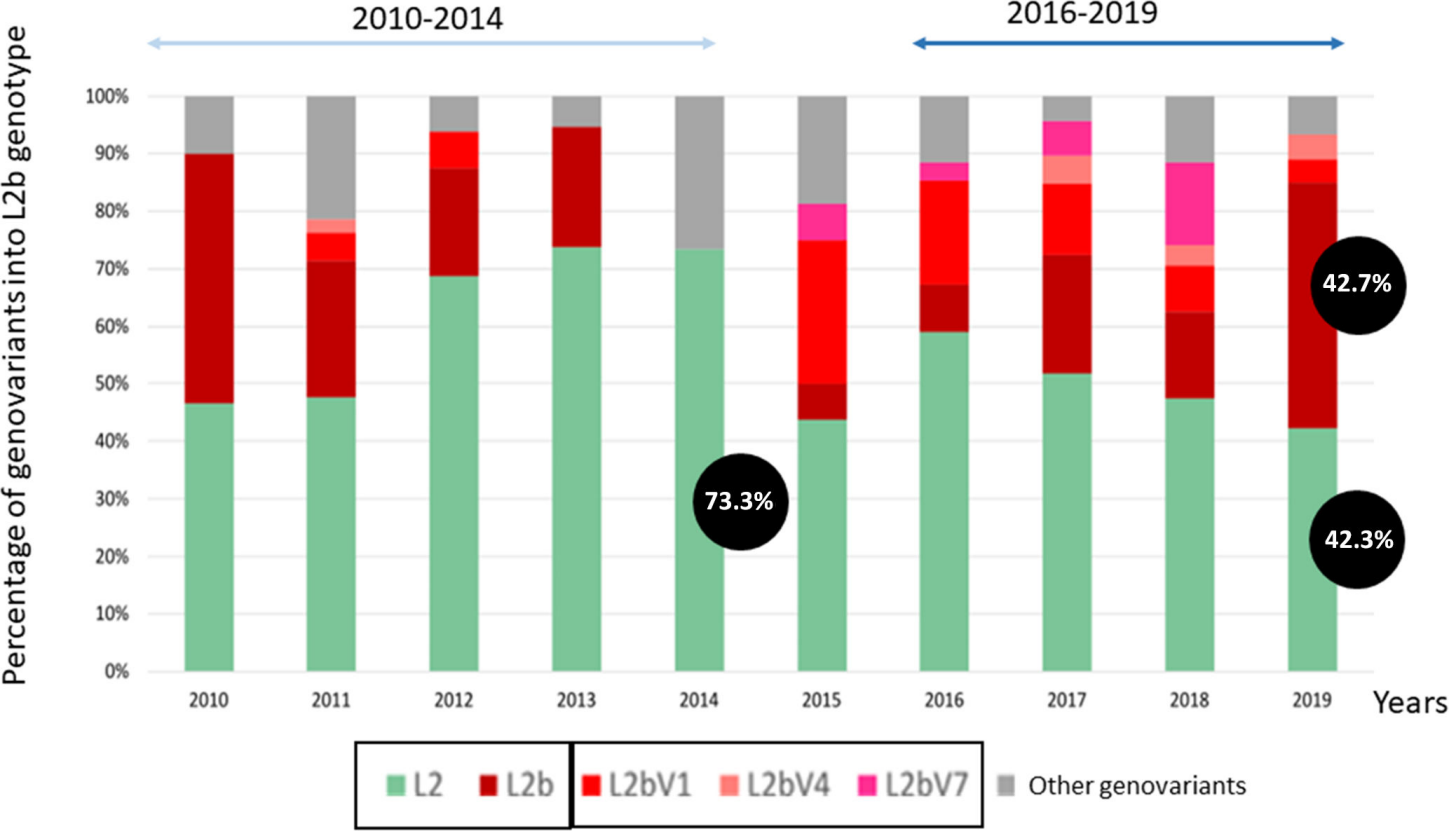
Distribution of *ompA* genovariants of lymphogranuloma venereum during the study period (2010–2019). The samples of the four participating centers are included (*n* = 803). The *ompA*-L2 genovariant was the most prevalent throughout all periods. However, there was a gradual phaseout of *ompA*-L2b and its evolved genovariants in favor of *ompA*-L2 and its genovariants (51.8%, 60%, 68.7%, and 82.3% in 2010, 2011, 2012, and 2013, respectively), while in 2016–2019, there was an inverse trend in which *ompA*-L2b and its evolved genovariants were progressively detected more frequently (31.6%, 44.4%, 46.8%, and 55.1% in 2016, 2017, 2018, and 2019, respectively).

We analyzed the distribution of genovariants found in the 2010–2014 and 2016–2019 periods outlined in the previous section. Figure 4 shows the phylogenetic relationship between the *ompA*-L genotypes described in Madrid during the two periods. The comparison between the 2010–2014 and 2016–2019 periods shows the evolution of the LGV epidemiological situation toward a more complex scenario characterized by an increasing number of genovariants. There were three main differences between the two periods: (i) During 2010–2014, there was a gradual phaseout of *ompA*-L2b and its evolved genovariants in favor of *ompA*-L2 and its evolved genovariants, while in 2016–2019, there was an inverse trend in which *ompA*-L2b and its evolved genovariants were progressively detected more frequently (a more detailed information in Fig. 5). (ii) Recombinant forms were detected in the second period. The first L2-D recombinant was detected in 2016 (1.7%) and, in successive years, the detection of these recombinants progressively increased [5 (2.7%), 14 (7%), and 11 (4.8%) in 2017, 2018, and 2019, respectively]. Three patterns were identified among the recombinant forms, with pattern 2 the most frequently detected (Table S3). (iii) The success of new genovariants in 2016–2019. The *ompA*-L2bV1 genovariant was reported during the first period at a proportion of 3.7%–6.2%. During the second period, however, the proportion reached 26.6% in 2016 and progressively decreased to 4.0% in 2019 (Table S2). The *ompA*-L2bV4 genovariant was found only in 2011 and in only one strain; in the second period, however, it was detected during the last 3 years in 4.0%–4.9%. Two other genovariants were detected: (i) *ompA*-L2bV7 which was detected in 2015, reaching 14.5% of cases in 2018 but surprisingly disappearing in 2019 and (ii) *ompA*-L2 and *ompA*-L2b carrying the Q75R, a genovariant detected in 2010–2011, which reached 13.3% in 2011 and then disappeared.

### Epidemiological and clinical characteristics associated with the main genovariants belonging to the L2b genotype

We reviewed 428 of the 730 medical records corresponding to patients infected with one of the five most prevalent genovariants; however, not all of the clinical and epidemiological data were always available. The clinical and epidemiological characteristics of the *ompA*-L genovariants are shown in Table 2. The participants’ median age was 34 years (IQR 29–41), and most were MSM (308/316; 97.5%). Most of the patients were from Spain (251/415; 60.5%) and Latin America (137/415; 33.0%). Asymptomatic patients accounted for 21% (71/340) of the study population. In patients carrying L genotypes, HIV infection (241/350) was decreasing from 84.3% to 66.2%. In addition, most of the patients with HIV infection had a well-controlled infection and undetectable viral load (134/181; 74%), reaching until 83.4% in the final 3 years of the study. Regarding hepatitis C virus (HCV), 15.6% (44/281) of the patients had viremia.

**TABLE 2:** Clinical and epidemiological characteristics of patients stratified by the genovariants detected (L2, L2b, L2bV1, L2bV4, and L2bV7)

	L2 (%)	L2b (%)	L2bV1 (%)	L2bV4 (%)	L2bV7 (%)	*P*
Symptoms/signs	*N* = 146	*N* = 126	*N* = 42	*N* = 26	*N* = 28	
Pain	66 (45.2)	72 (57.1)	21 (50.0)	12 (46.2)	13 (46.4)	0.255
Purulent discharge	50 (34.2)	36 (28.6)	9 (21.4)	5 (19.2)	9 (32.1)	0.233
Bleeding	50 (34.2)	49 (38.9)	13 (30.9)	6 (23.1)	11 (39.3)	0.429
Edema	46 (31.5)	50 (39.7)	18 (42.9)	9 (34.6)	11 (39.3)	0.409
Ulcer	11 (7.5)	15 (11.9)	5 (11.9)	3 (11.5)	3 (10.7)	0.573
None	37 (25.3)	20 (15.9)	10 (23.8)	4 (15.4)	5 (17.9)	0.225
HIV serological status	*N* = 144	*N* = 136	*N* = 44	*N* = 26	*N* = 33	
Positive	108 (75)	84 (61.8)	37 (84.1)	12 (46.2)	22 (66.7)	0.001
HCV serological status	*N* = 97	*N* = 118	*N* = 36	*N* = 26	*N* = 30	
Positive	21 (21.6)	16 (13.6)	5 (13.9)	2 (7.7)	4 (13.3)	0.273
	*N* = 101	*N* = 118	*N* = 40	*N* = 25	*N* = 27	
Concomitant STI	46 (45.5)	54 (45.8)	16 (40)	12 (48.0)	10 (37.0)	0.911
Syphilis	17 (16.8)	15 (12.7)	6 (15)	3 (12.0)	0	0.852
*N. gonorrhoeae*	30 (29.7)	35 (29.7)	12 (30)	8 (32.0)	8 (29.3)	0.996
*C. trachomatis*	6 (5.9)	5 (4.2)	0	2 (8.0)	1 (3.7)	0.6
Others (HPV, HSV-2)	1 (0.9)	10 (8.5)	0	8 (32.0)	2 (7.4)	0.017
	*N* = 81	*N* = 108	*N* = 26	*N* = 25	*N* = 23	
Previous STIs	68 (83.9)	95 (88.0)	19 (73.1)	24 (96.0)	21 (91.3)	0.114
Syphilis	54 (66.7)	69 (63.9)	14 (53.8)	12 (48.0)	18 (78.3)	0.292
*N. gonorrhoeae*	37 (45.7)	50 (46.3)	10 (38.5)	21 (84.0)	11 (47.8)	0.003
*C. trachomatis*	20 (24.7)	22 (20.4)	5 (19.2)	9 (36.0)	6 (26.1)	0.376
LGV	11 (13.6)	17 (15.7)	1 (3.8)	3 (12.0)	0	0.499
Others (HPV, HSV-2)	37 (24.7)	17 (15.7)	4 (15.4)	5 (20.0)	2 (8.7)	0.462
Patient origin	*N* = 194	*N* = 144	*N* = 51	*N* = 26	*N* = 31	
Spain	112 (57.7)	92 (63.9)	35 (68.6)	12 (46.2)	19 (61.3)	0.175
Latin America	68 (35.1)	45 (31.3)	13 (25.5)	11 (42.3)	12 (38.7)	0.409
Other European countries	11 (5.7)	4 (2.8)	3 (5.9)	2 (7.7)	0	0.394
Other	3 (1.5)	3 (2.1)	0	1 (3.8)	0	0.474
Drug user	*N* = 76	*N* = 101	*N* = 26	*N* = 26	*N* = 22	
	38 (50.0)	67 (66.3)	16 (61.5)	16 (61.5)	16 (72.8)	0.179
Sexual behavior	*N* = 121	*N* = 130	*N* = 40	*N* = 25	*N* = 25	
MSM	118 (97.5)	125 (96.2)	40 (100)	24 (96.0)	24 (96.0)	0.701
Bisexual	1 (0.8)	4 (3.1)	0	1 (4.0)	1 (4.0)	0.379
MSW	2 (1.7)	0	0	0	0	0.355
Male sex workers	12 (9.9)	11 (8.5)	4 (10.0)	2 (8.0)	2 (8.0)	0.972

Differing patterns were observed by genotype: *ompA*-L2bV1 was more associated with Spaniards than with Latin Americans (68.6%–25.5%), while *ompA*-L2bV4 was associated at a similar rate in the two groups, but these differences were not statistically significant. Asymptomatic infections were more associated with *ompA*-L2 (25.3%) and *ompA*-L2bV1 (23.8%) than with *ompA*-L2b (15.3%) or ompA-L2bV4 (15.4%). HIV infection was more frequently associated with the *ompA*-L2 (75%) and *ompA*-L2bV1 (84.1%) genovariants than with *ompA*-L2b (61.8%) or *ompA*-L2bV4 (46.2%), while *ompA*-L2bV7 (66.7%) had an intermediate position. These differences were statistically significant (*P* = 0.001), as were other differences in concomitant or previous STIs. Again, *ompA*-L2b and *ompA*-L2bV4 were associated with concomitant HPV and HSV-2 infections (*P* < 0.02) and previous *N. gonorrhoeae* infections (*P* = 0.003). Similar results were observed for HCV seroprevalence, which was more common among those infected with *ompA*-L2 (21.6%) and *ompA*-L2bV1 (13.9%) compared with *ompA*-L2b (12.6%) or ompA-L2bV4 (7.7%). The results suggest that these genovariants spread in distinct sexual networks. In fact, *ompA*-L2 and *ompA*-L2bV1 might share a similar sexual network, whereas *ompA*-L2b and *ompA*-L2bV4 are spreading in a different network.

## DISCUSSION

In this study, we first analyzed the trends of LGV infections diagnosed in a community healthcare center specialized in STIs and attended mostly by MSM over a 10-year period (2010–2019). In 2016, when the number of detected infections increased dramatically in this center, we extended the study to three additional community and hospital centers with different profiles of STI exposure. This study covers one of the longest periods and includes the largest number of detected LGV infections (>1,000), including the partial sequencing of the *pmpH* gene, which correctly identifies the *C. trachomatis* genotype. More than 800 cases were sequenced as *ompA* and belonged to the L2b genotype, which provides insight into the emergence, selection, and dispersion of genovariants in a single large city.

The current European LGV epidemic started in the Netherlands and was associated with a single novel L2b genotype (4). The co-circulation of two main genovariants based on the *pmpH* and *ompA* genes, corresponding to *ompA*-L2 and *ompA*-L2b, was initially reported in Spain (10). Several European countries have progressively observed this co-circulation (11, 16). An international collaborative study demonstrated (through whole-genome sequencing) that all variants belonged to the same L2b genotype (12). Since then, just one genotype has been considered primarily responsible for the European LGV epidemic (although spot detections of non-*ompA*-L2b have been reported). The sequencing of *pmpH* and *ompA* has become a popular technique in LGV epidemiology. *PmpH* guarantees accurate genotype assignment, while the *ompA* gene is subject to the greatest selective pressure (positive selection), with an overrepresentation of genomic mutations. Therefore, both genes could help detect a rapid evolution based on the selection and diversification of *ompA* L genovariants (12), as occurred in our study when *ompA* mutations were found in domains I–IV, which are surface-exposed domains involved in adhesion and immune response (23). Consequently, this strategy enables a quick understanding of LGV’s evolutionary drift during an outbreak.

During the study period, the screening strategies and methods improved in all diagnostic centers, resulting a higher number of recorded *C. trachomatis* infections and making it difficult to estimate significant changes in the prevalence. Our results suggest that the improved STI screening detected more infections but that there was an actual increase in LGV infections. In the STI1 center (mainly attended by MSM), however, the LGV/*C. trachomatis* ratio increased from 6.8% to 19.3%, suggesting a marked surge in LGV infections among those predominantly receiving care at STI1. The number of new diagnoses of *C. trachomatis* infections also increased more than the increase in detection. At the STI2 center, the diagnoses of LGV compared with those of *C. trachomatis* slightly increased (2.0%–3.6%), also suggesting an increase in LGV transmission. Nevertheless, the number of new diagnoses of *C. trachomatis* infections remained proportional to the improvement in screening. In the hospital setting, however, the number of samples for STI screening also increased, but the LGV/*C. trachomatis* ratio remained nearly constant, suggesting that the detected LGV cases were related to improved screening rather than to a greater spread of LGV in the population served by the hospitals. These findings suggest that LGV infections are commonly found among MSM with an increased risk of contracting STIs. In these individuals, we also observed an alarmingly rapid rise in such infections. Other populations also experienced a progressive increase in LGV infections, indicating that *C. trachomatis* genotyping should be extended to all individuals.

The temporal analysis of STI1 revealed an acceleration in LGV diagnoses during the 2015–2019 period, with almost double the diagnoses compared with the previous period (2010–2014); similar results were observed in the UK (24). Several factors might have contributed to this epidemiological shift. Although not an objective of this study, there was a recent increase in the use of sex-seeking mobile phone applications (25) and of psychoactive drugs in the context of sexual relations, which gained popularity in Spain from 2015 (26), and the fact that the Community of Madrid has the highest rate of psychoactive drug consumption in the Spain (27). All of these factors could certainly have played a role in the epidemiology of STIs (including LGV) because highly interconnected sexual exchange networks that act as transmission clusters can annually affect the local epidemiology, as we observed with the selection of certain genovariants. Another explanation could be related to the re-introduction of LGV to Spain, given that when the pattern of mutations in *ompA* is compared with that of successive years, the mutations detected in 2010–2014 differ from those in 2016–2019. Moreover, we observed a progressive slowdown of the epidemic in 2010–2014. The re-introduction hypothesis needs to be confirmed with the sequencing of LGV strains from those countries with a strong cultural relationship with Spain.

The current data reveal that, since 2015, at least five *ompA*-L2b genovariants have been responsible for the LGV epidemic in Madrid, showing an epidemiological scenario that is becoming progressively more complex. Spain could therefore have the highest LGV genotype diversity in Europe, as was suspected in the previous international collaboration on LGV in Europe (23). The L2bV1 and L2bV4 genotypes have been identified in other European countries such as France (24), Italy (12), Austria, and the UK (28), but in each case, the first detected case was in Spain (15, 23); the L2bV7 genotype has been reported only in Spain (12). Our series could indicate an expected evolution of LGV epidemics in other European countries with a higher rate of underdiagnosis.

The high diversification of *ompA*-L2b genovariants observed in our study was the result of mutational and recombinational events. High prevalence and high transmission rates, particularly in the sexual encounter hot points of MSM, imply denser bacterial populations and consequently higher chances of selecting point-mutated genovariants. Recombination is also proportional to the absolute bacterial density, facilitating the coexistence of *C. trachomatis* genovariants, possibly in mixed infections, as observed in our previous study (29). The increase in recombinant forms detected in the last 3 years is a new concern that confirms our worst predictions with respect to the maintenance of LGV in Spain. Our study reveals the actual increased risk of *C. trachomatis* generating new variants (30), which could be endowed with distinct tropisms, exploiting new ecological niches and spreading to other population groups. Efforts should, therefore, be undertaken to strengthen screening and surveillance programs, especially in the current situation with the continuous increase in STI infections and the post-pandemic SARS-CoV-2 period in which numerous screening programs were interrupted (31).

Clinical and epidemiological data obtained from patients infected with various *ompA*-L2b genovariants (including *ompA*-L2) reveal how patients infected by *ompA*-L2 and *ompA*-L2bV1 genovariants share clinical and epidemiological data, which differ from those exhibited by patients infected with *ompA*-L2b and *ompA*-L2bV4, suggesting different sexual networks. Probably, the most relevant association is HIV serostatus (*P* = 0.001) between the two networks, which is related to the serosorting phenomenon (a strategy that consists of selecting sexual partners of the same HIV serostatus). According to this interpretation, serosorting could have had an impact on the dissemination of *ompA*-L2b genovariants. In early publications on the European LGV epidemic, the association between LGV and HIV was worryingly high; however, recently published studies have detected a reduction in the association between LGV and HIV over time (5, 24) to approximately 64%. Our study has a similar finding compared with previous studies in Madrid in which 75% of LGV diagnoses occurred in patients with HIV. In the present study, 68% of the new LGV diagnoses were detected in the patients with HIV. According to the serosorting phenomenon, the HIV-LGV relationship could be affected by the number of screened individuals belonging to one or the other network. In any case, the introduction of pre-exposition prophylaxis and the increase in STI screening programs in these patients could drastically accelerate the rate of new LGV diagnoses.

This study has several limitations. We only analyzed the *ompA* and *pmpH* genes and, although the *ompA* gene is one of the most variables in *C. trachomatis*, our epidemiological evaluation might be limited. Moreover, the detection of the *ompA* gene associated with the L2b genotype does not imply that the entire genome corresponds to L2b (12). Other limitations include the disproportion in the *ompA* gene sequenced during the first and second study period (34.2%; 120/351 vs 83.3%; 683/820) and the limited clinical and epidemiological data.

In conclusion, our study demonstrates that improved screening and systematic genotyping of *C. trachomatis* resulted in a much higher number of cases and greater etiological genotype diversity in LGV infections. LGV infections continue to mainly affect the MSM population, but screening should be improved in all healthcare settings. Molecular characterization based on *pmpH* and *ompA* showed a temporal diversification of LGV and a progressive trend toward a more complex epidemiological scenario that should be analyzed in depth in future research.

## Data Availability

The authors affirm that all supporting data, code, and protocols have been provided within the article or through supplemental data files. The sequences obtained in this study were deposited in GenBank under the following accession numbers: OR264840 to OR265488.

## SUPPLEMENTAL MATERIAL

Figure S1 spectrum.02855-23-s0001.png Flowchart of the experimental design with results from the STI1.

Table S1 spectrum.02855-23-s0002.docx Primers, probes, and conditions used for detection of L-genotypes and *pmpH* and *ompA* sequencing.

Table S2 spectrum.02855-23-s0003.docx Summary of the *ompA* sequenced L-variants and the amino acid changes referred to the L2/434/Bu strain (AM884176).

Table S3 spectrum.02855-23-s0004.docx Patterns of recombinant forms detected in the final years (2016-2019).

ASM does not own the copyrights to Supplemental Material that may be linked to, or accessed through, an article. The authors have granted ASM a non-exclusive, world-wide license to publish the Supplemental Material files. Please contact the corresponding author directly for reuse.
