# Supplementary figures and images for: Rapid expansion of lymphogranuloma venereum infections with fast diversification and spread of Chlamydia trachomatis L genovariants
Source: Microbiol Spectr. 2023 Dec 14;12(1):e02855-23. doi: 10.1128/spectrum.02855-23 (PMC10783107; doi:10.1128/spectrum.02855-23)

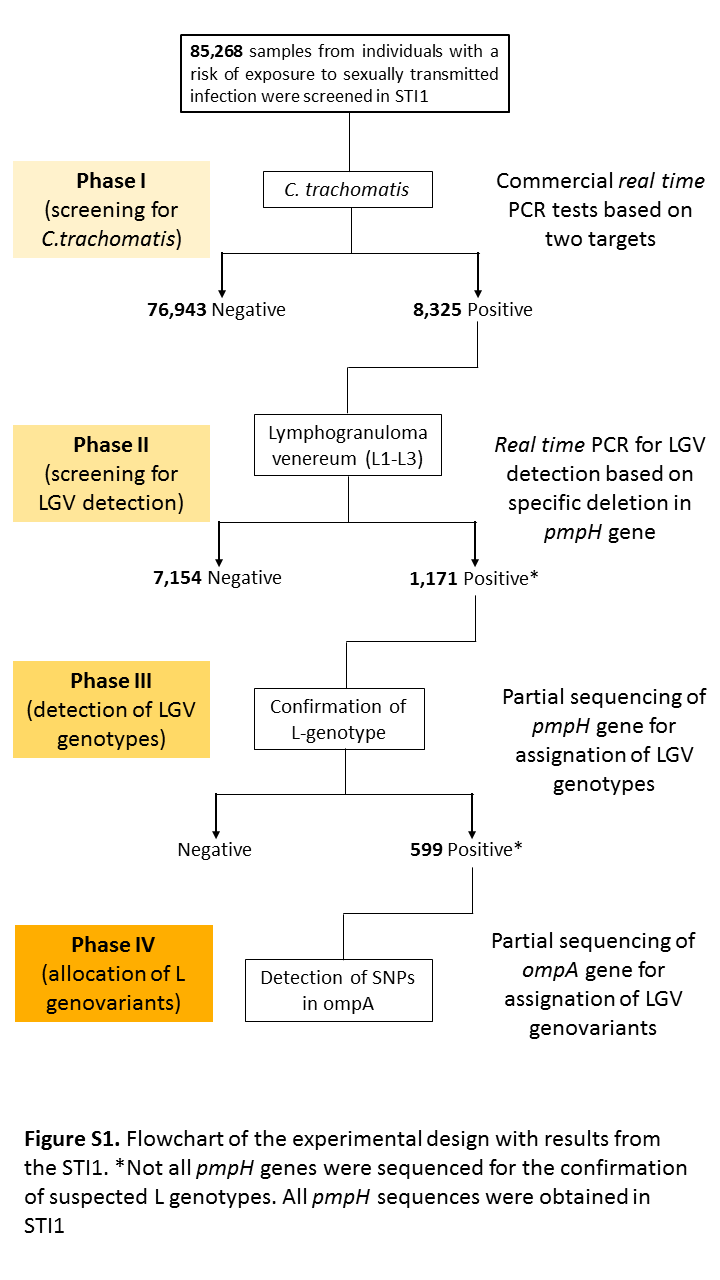

Supplement: Figure S1 — Flowchart of the experimental design with results from the STI1. [file spectrum.02855-23-s0001.png]
