# Supplementary material for: Rapid expansion of lymphogranuloma venereum infections with fast diversification and spread of Chlamydia trachomatis L genovariants
Source: Microbiol Spectr. 2023 Dec 14;12(1):e02855-23. doi: 10.1128/spectrum.02855-23 (PMC10783107; doi:10.1128/spectrum.02855-23)
Supplement: Table S1 — Primers, probes, and conditions used for detection of L-genotypes and pmpH and ompA sequencing. [file spectrum.02855-23-s0002.docx]

| **Screening of L-genotypes** | | | |
| --- | --- | --- | --- |
| Primers/probe | Gene | Sequence (5´-3´) | Conditions |
| LGV_F | *pmpH* | CTGTGCCAACCTCATCATCAA | 95ºC⎯1'  95ºC⎯30´´  40x  60ºC⎯30´´ |
| LGV_R |  | AGACCCTTTCCGAGCATCACT |  |
| LGV_probe |  | FAM-CCGCCTGCTCCAACAGTTAGTGATG-BHQ1 |  |
| ***pmpH* sequencing** | | | |
| Primers | Gene | Sequence (5´-3´) | Conditions |
| *pmpH*_F | *pmpH* | AAACCGTGAGTATTTCCGGA | 94ºC⎯12'  94ºC⎯30´´  40x  57ºC⎯30´´  72ºC⎯1'  72ºC⎯10´ |
| *pmpH*_R |  | ATATGCCTCCTTCATTGTCTT |  |
| ***ompA* sequencing** | | | |
| Primers | Gene | Sequence (5´-3´) | Conditions |
| *ompA*_F | *ompA* | AACCAAGCCTTATGATCGACGGAAT | 94ºC⎯12'  94ºC⎯30´´  40x  58ºC⎯30´´  72ºC⎯1'  72ºC⎯10´ |
| *ompA*_R |  | CAATACCGCAAGATTTTCTAGATTTCA |  |

**Table S1.** Primers, probes, and conditions used for detection of L-genotypes and *pmpH* and *ompA* sequencing.
