# Supplementary material for: Rapid expansion of lymphogranuloma venereum infections with fast diversification and spread of Chlamydia trachomatis L genovariants
Source: Microbiol Spectr. 2023 Dec 14;12(1):e02855-23. doi: 10.1128/spectrum.02855-23 (PMC10783107; doi:10.1128/spectrum.02855-23)

**Table S2.** Summary of the *ompA* sequenced L-variants and the amino acid changes referred to the L2/434/Bu strain (AM884176), classified by year and the percentage of each mutation among the sequences obtained each year. *The N162S, which helps differentiate between L2 and L2b, is not included. ^(1)^ The RF in the last column represents the percentage of recombinant forms.


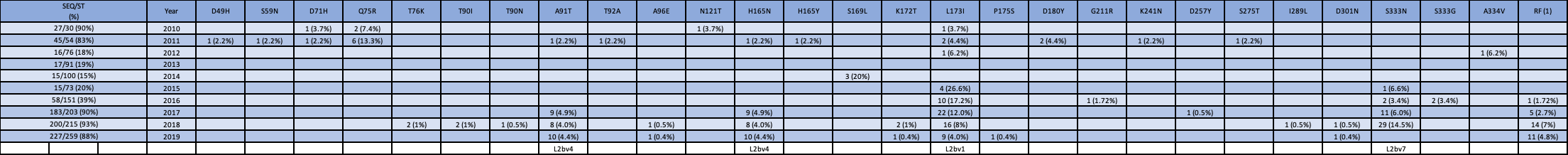

Supplement: Table S2 — Summary of the ompA sequenced L-variants and the amino acid changes referred to the L2/434/Bu strain (AM884176). [file spectrum.02855-23-s0003.docx]
