# Supplementary material for: Rapid expansion of lymphogranuloma venereum infections with fast diversification and spread of Chlamydia trachomatis L genovariants
Source: Microbiol Spectr. 2023 Dec 14;12(1):e02855-23. doi: 10.1128/spectrum.02855-23 (PMC10783107; doi:10.1128/spectrum.02855-23)

**Table S3**. Patterns of recombinant forms detected in the final years (2016–2019). The position S169- shows a deletion. In pattern 3, the A166S is a new point mutation with respect to the previously observed mutation (A166K).


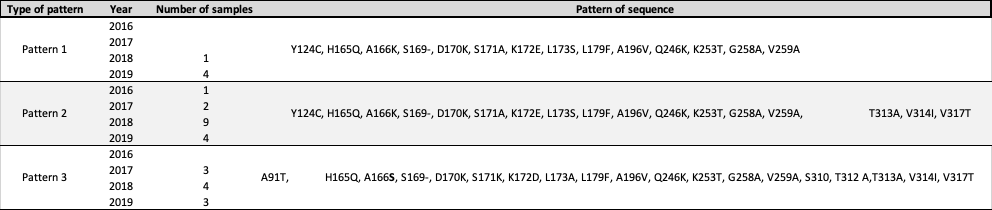

Supplement: Table S3 — Patterns of recombinant forms detected in the final years (2016-2019). [file spectrum.02855-23-s0004.docx]
